# Supplementary material for: Structural basis of ligand binding modes of human EAAT2
Source: Nat Commun. 2022 Jun 9;13:3329. doi: 10.1038/s41467-022-31031-x (PMC9184463; doi:10.1038/s41467-022-31031-x)
Supplement: Supplementary file 1 — Supplementary Information [file 41467_2022_31031_MOESM1_ESM.pdf]

Supplementary information

Structural basis of ligand binding modes of human EAAT2

Zhenglai Zhang[1,2]*, Huiwen Chen[1,2]*, Ze Geng[3,4]*, Zhuoya Yu[2,5,6], Hang Li[2,6], Yanli Dong[2], Hongwei Zhang[2,6], Zhuo Huang[3,4]**, Juquan Jiang[1]**, Yan Zhao[2,5,6]**

[1] Department of Microbiology and Biotechnology, College of Life Sciences, Northeast Agricultural University, No. 600 Changjiang Road, Xiangfang District, Harbin 150030, China

[2] National Laboratory of Biomacromolecules, CAS Center for Excellence in Biomacromolecules, Institute of Biophysics, Chinese Academy of Sciences, Beijing 100101, China

[3] State Key Laboratory of Natural and Biomimetic Drugs, Department of Molecular and Cellular Pharmacology, School of Pharmaceutical Sciences, Peking University Health Science Center, Beijing, 100191, China

[4] IDG/McGovern Institute for Brain Research, Peking University, Beijing, 100871, China

[5] State Key Laboratory of Brain and Cognitive Science, Institute of Biophysics, Chinese Academy of Sciences, 15 Datun Road, Beijing, 100101, China

[6] College of Life Sciences, University of Chinese Academy of Sciences, Beijing 100049, China

* These authors contributed equally

** Correspondence emails: zhaoy@ibp.ac.cn (Y.Z.), jjqdainty@163.com (J.J.), and huangz@hsc.pku.edu.cn (Z.H.)

Supplementary Figures

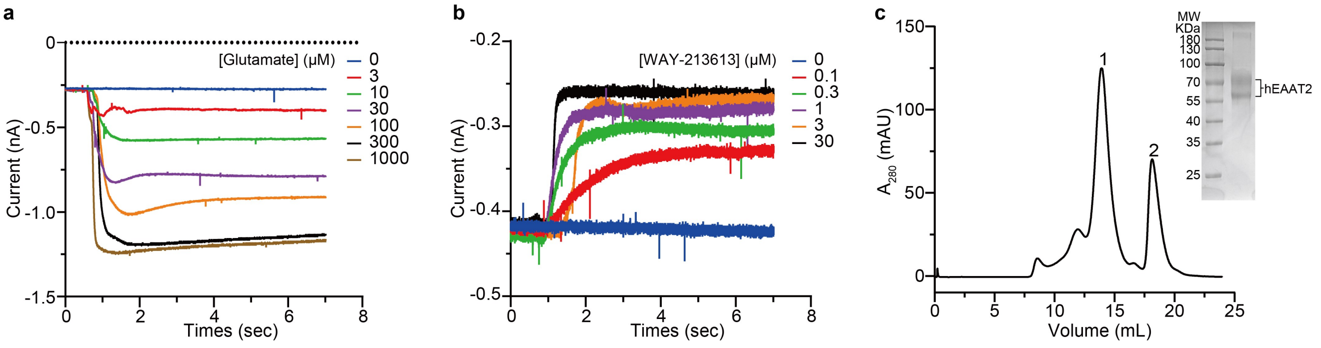

Supplementary Fig. 1 Functional characterization and purification of wild-type hEAAT2 samples. a Current traces obtained when 3 μM, 10 μM, 30 μM, 100 μM, 300 μM, 1000 μM glutamate are applied to the hEAAT2-expressed HEK293T cells. **b** Current traces obtained when 0.1 μM, 0.3 μM, 1 μM, 3 μM, 30 μM WAY-213613 are applied to hEAAT2-expressed HEK293T cells. In the figures **a** and **b**, all experiments were executed at 0 mV and the experimental procedure was the same as described in the method. **c** The purified hEAAT2 samples were subjected to size exclusion chromatography analysis (Superose 6 Increase 10/300 GL) after the pre-incubation with a PreScission protease for the complex with glutamate or WAY-213613. The peak 1 fraction was concentrated for the preparation of Cryo-EM samples. The peak 2 fraction is indicative of the cleaved GFP. Inset: SDS-PAGE of the hEAAT2 samples for Cryo-EM analysis. hEAAT2 was separated into two bands due to glycosylation. All experiments were independently executed at least three times with the similar results.

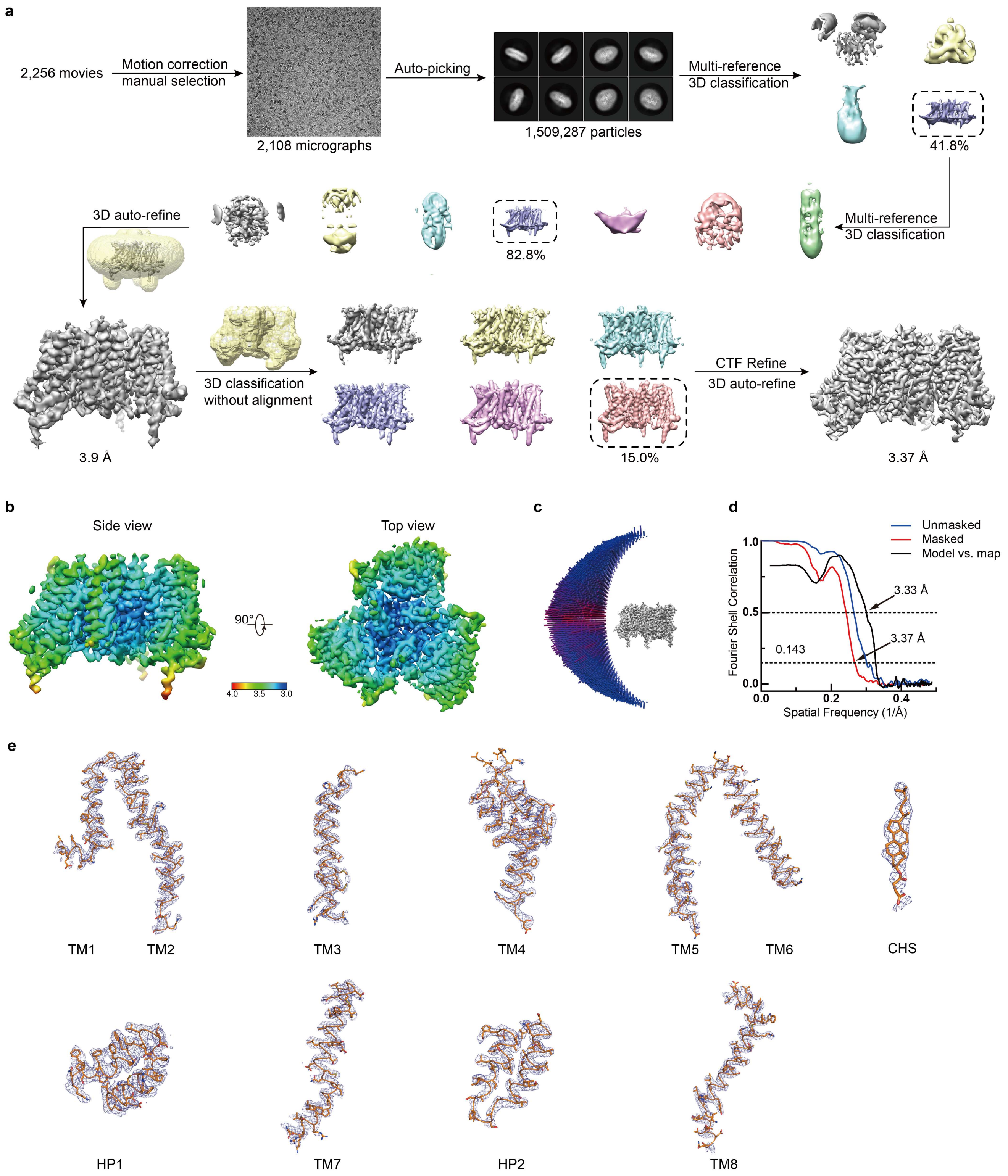
a
2,256 movies
Motion correction
manual selection
2,108 micrographs
Auto-picking
1,509,287 particles
Multi-reference
3D classification
41.8%
Multi-reference
3D classification
82.8%
3D auto-refine
3.9 Å
3D classification
without alignment
15.0%
CTF Refine
3D auto-refine
3.37 Å
b
Side view
Top view
90°
4.0
3.5
3.0
c
d
Unmasked
Masked
Model vs. map
3.33 Å
3.37 Å
0.143
Fourier Shell Correlation
Spatial Frequency (1/Å)
e
TM1
TM2
TM3
TM4
TM5
TM6
CHS
HP1
TM7
HP2
TM8

Supplementary Fig. 2 Cryo-EM data processing of the hEAAT2^{Glu} complex. a Flow chart of cryo-EM data processing. 2,256 movie stacks were collected on a 300-kV Titan Krios using a K2 summit direct electron detector, and 2,108 micrographs were used for particle picking using software Auto-picking. 1,509,287 particles were then submitted to several rounds of 3D classification, and CTF and 3D-auto refinement using C3 symmetry. A 3.4 Å resolution map of hEAAT2^{Glu} was obtained. Masks used for 3D refinement were shown as transparent surfaces alongside the arrows. **b** hEAAT2^{Glu} density maps colored by the local resolution. **c** Angular distribution of the particles included in the final 3D reconstruction. The length and color of each cylinder are indicative of the number of particles in the designated orientation (long and red: high number of particles; short and blue: low number of particles). **d** Fourier Shell Correlation (FSC) curves of the final refined unmasked (blue) and masked (red) map, and the refined model versus the map of hEAAT2^{Glu} for cross-validation (black). **e** Representative EM maps for hEAAT2^{Glu}.

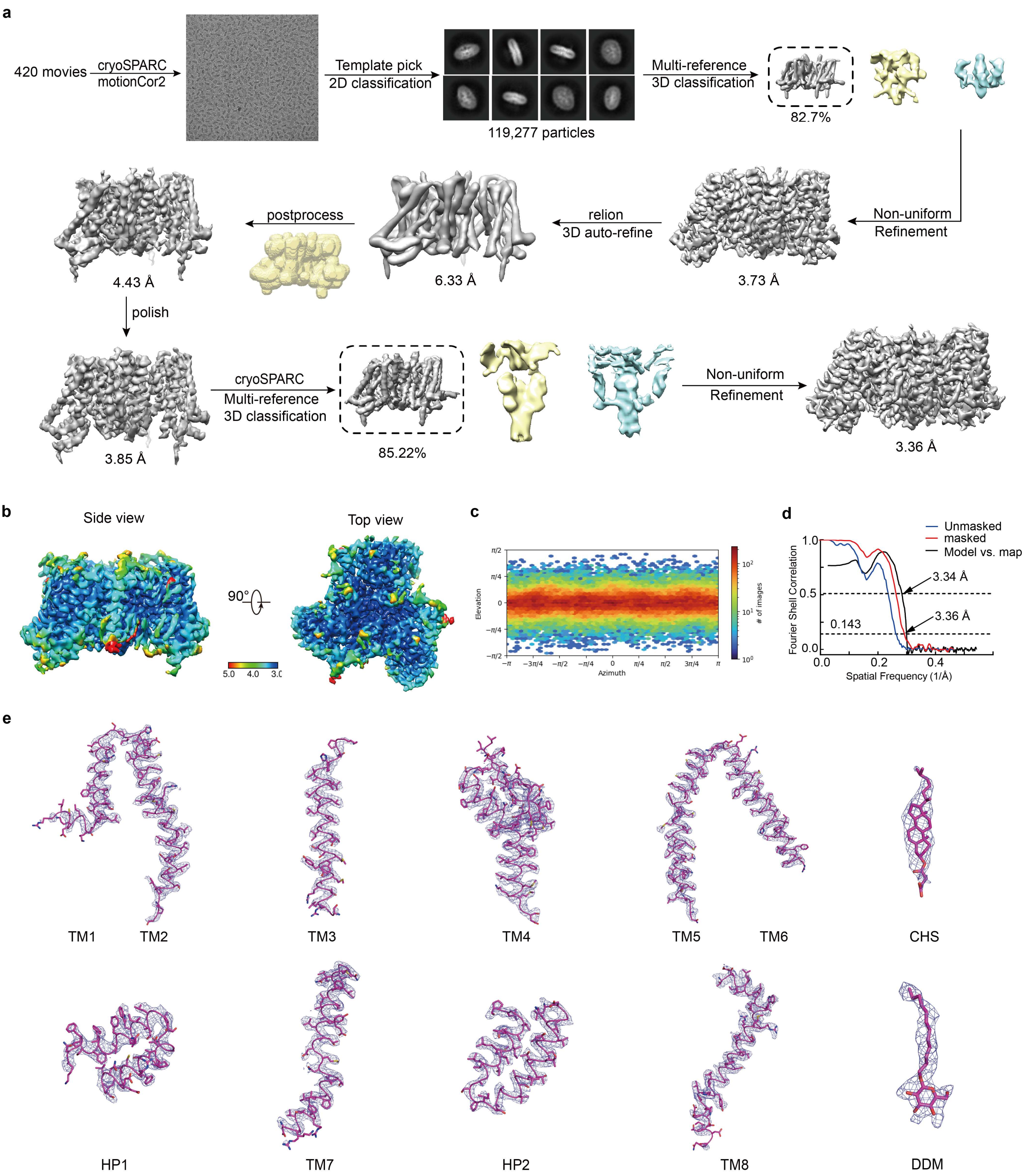
a
420 movies
cryoSPARC
motionCor2
Template pick
2D classification
119,277 particles
Multi-reference
3D classification
82.7%
Non-uniform
Refinement
3.73 Å
relion
3D auto-refine
6.33 Å
postprocess
4.43 Å
polish
3.85 Å
cryoSPARC
Multi-reference
3D classification
85.22%
Non-uniform
Refinement
3.36 Å
b
Side view
Top view
90°
5.0
4.0
3.0
c
Elevation
Azimuth
# of images
d
Unmasked
masked
Model vs. map
Fourier Shell Correlation
Spatial Frequency (1/Å)
3.34 Å
3.36 Å
0.143
e
TM1
TM2
TM3
TM4
TM5
TM6
CHS
HP1
TM7
HP2
TM8
DDM

Supplementary Fig. 3 Cryo-EM data processing of the hEAAT2^W complex. a Flow chart of cryo-EM data processing. 465 movie stacks were collected on a 300-kV Titan krios using a K2 Summit direct electron detector, and 420 micrographs were used for particle picking using Template picker in cryoSPARC. The extracted 233,008 particles were then submitted to 2D classification, 3D Heterogeneous Refinement with C3 symmetry, and a 3.7 Å map obtained. Particles were re-extracted in RELION-3.1 for further improvement of the particle quality, and then the polished particles were imported to cryoSPARC and subjected to 3D Heterogeneous Refinement with C3 symmetry imposed. Finally, a 3.4 Å resolution map of hEAAT2^W was obtained. Mask used for postprocessing was shown as transparent surfaces alongside arrow. **b** hEAAT2^W density maps colored by the local resolution. **c** Angular distribution of the particles included in the final 3D reconstruction using cryoSPARC. **d** Fourier Shell Correlation (FSC) curves of the final refined unmasked (blue) and masked (red) map, and the refined model versus the map of hEAAT2^W for cross-validation (black). **e** Representative EM maps for hEAAT2^W.

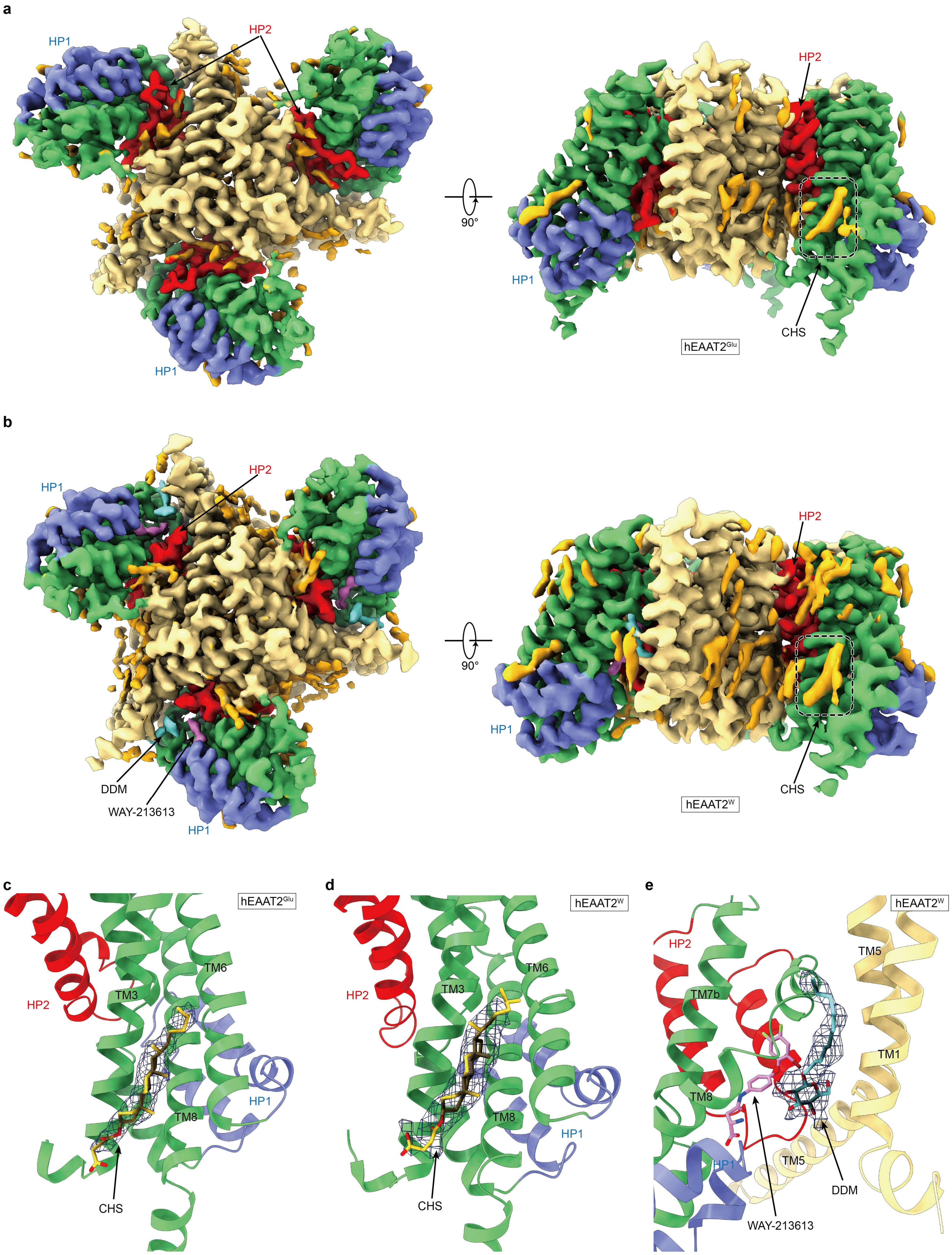

a
HP1
HP2
HP1
90°
HP2
HP1
CHS
hEAAT2Glu
b
HP1
HP2
DDM
WAY-213613
HP1
90°
HP2
HP1
CHS
hEAAT2W
c
hEAAT2Glu
HP2
TM3
TM6
HP1
TM8
CHS
d
hEAAT2W
HP2
TM3
TM6
TM8
HP1
CHS
e
hEAAT2W
HP2
TM7b
TM5
TM1
TM8
HP1
TM5
DDM
WAY-213613

Supplementary Fig. 4 Cryo-EM maps of the hEAAT2^{Glu} and hEAAT2^{W} complexes with the associated lipid molecules. **a** and **b** Cryo-EM maps of the hEAAT2^{Glu} and hEAAT2^{W} complexes. The homotrimer is viewed from the cytoplasm (left panel) and membrane plane (right panel), respectively. The scaffold domain and the transport domain are colored in wheat and green, respectively. HP1 and HP2 are colored in blue and red, respectively. CHS is highlighted in a dashed-line rectangular box. The densities of WAY-213613 and DDM are colored in pink and cyan, respectively. Lipid densities observed around the complex are highlighted in orange. **c** and **d** Densities of CHS in the hEAAT2^{Glu} and hEAAT2^{W} complexes, respectively. CHS is colored in yellow and the density is shown as mesh. **e** Density of DDM in the hEAAT2^{W} complex. DDM is colored in cyan and the density of DDM is shown as mesh.

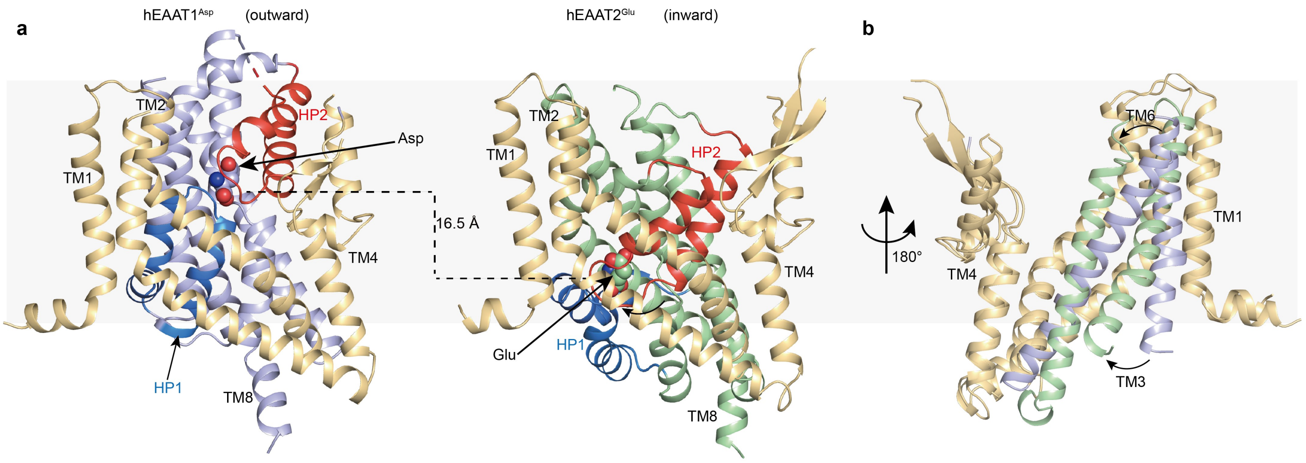

Supplementary Fig. 5 Structural comparison between the inward-facing hEAAT2^{Glu} and the outward-facing hEAAT1^{Asp} (PDB ID: 5LLU). a Conformational change between the transport domain of the inward-facing hEAAT2^{Glu} (pale green) and that of the outward-facing hEAAT1^{Asp} (light blue), using the scaffold domain (wheat) as a reference. The HP1 and HP2 are colored in blue and red, respectively. The substrate of the hEAAT1^{Asp} (left panel) and that of the hEAAT2^{Glu} (right panel) are shown as colored spheres. **b** Superimposed structures of the inward-facing hEAAT2^{Glu} (pale green) and the outward-facing hEAAT1^{Asp} (light blue).

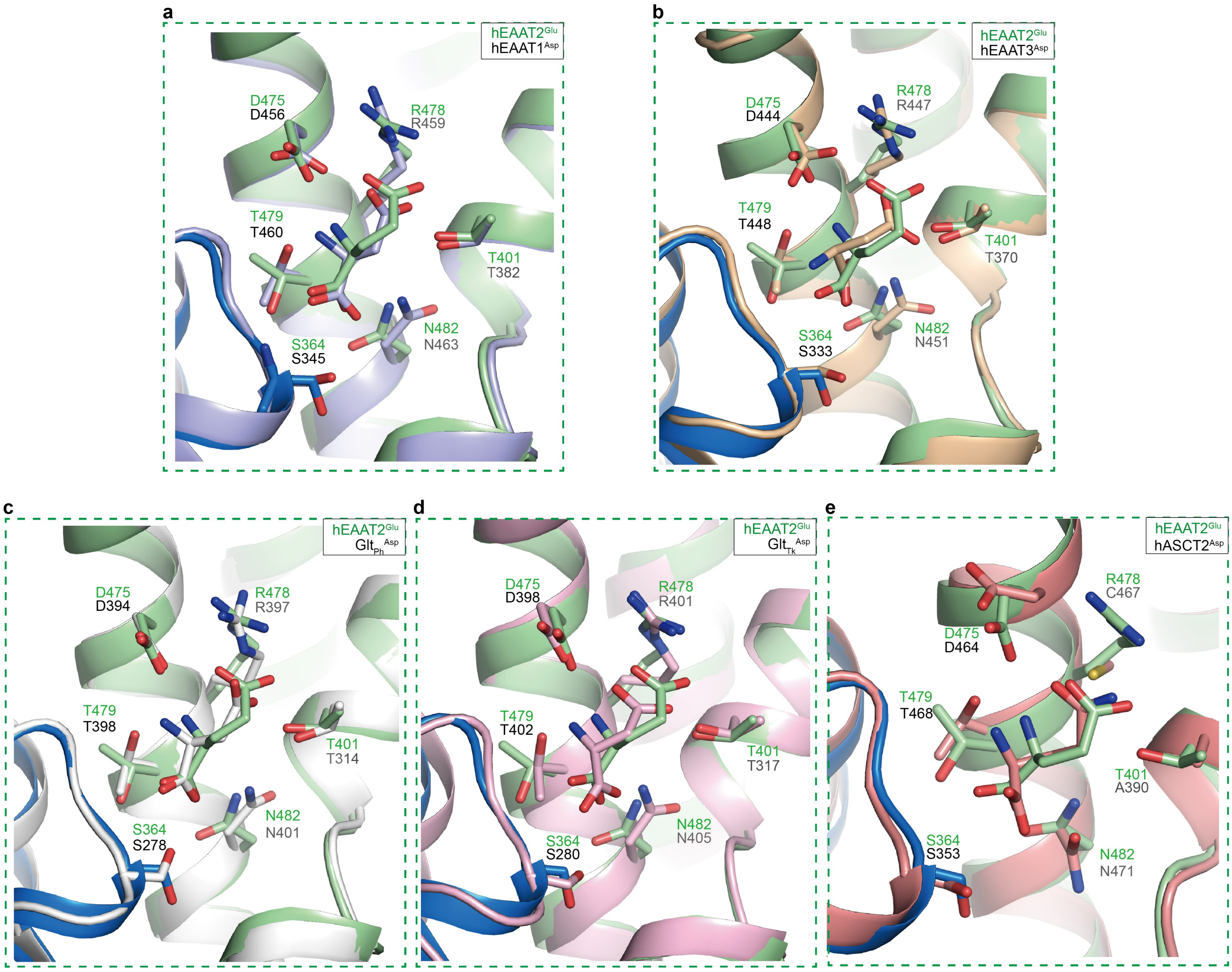

Supplementary Fig. 6 Structural comparison of the transport domains between hEAAT2^{Glu} complex with other substrate-bound structures. a Structural comparison of the transport domains between hEAAT2^{Glu} (pale green) and hEAAT1^{Asp} (PDB ID: 5LLU, light blue). **b** Structural comparison of the transport domains between hEAAT2^{Glu} (pale green) and hEAAT3^{Asp} (PDB ID: 6X2Z, wheat). **c** Structural comparison of the transport domains between hEAAT2^{Glu} (pale green) and Glt$_{Ph}^{Asp}$ (PDB ID: 6X15, white). **d** Structural comparison of the transport domains between hEAAT2^{Glu} (pale green) and Glt$_{Tk}^{Asp}$ (PDB ID: 6R7R light pink). **e** Structural comparison of the transport domains between hEAAT2^{Glu} (pale green) and hASCT2^{Asp} (PDB ID: 6GCT, salmon). In **a-e**, the HP1 tip of hEAAT2^{Glu} is highlighted in blue to be differentiated from those of other homologs.

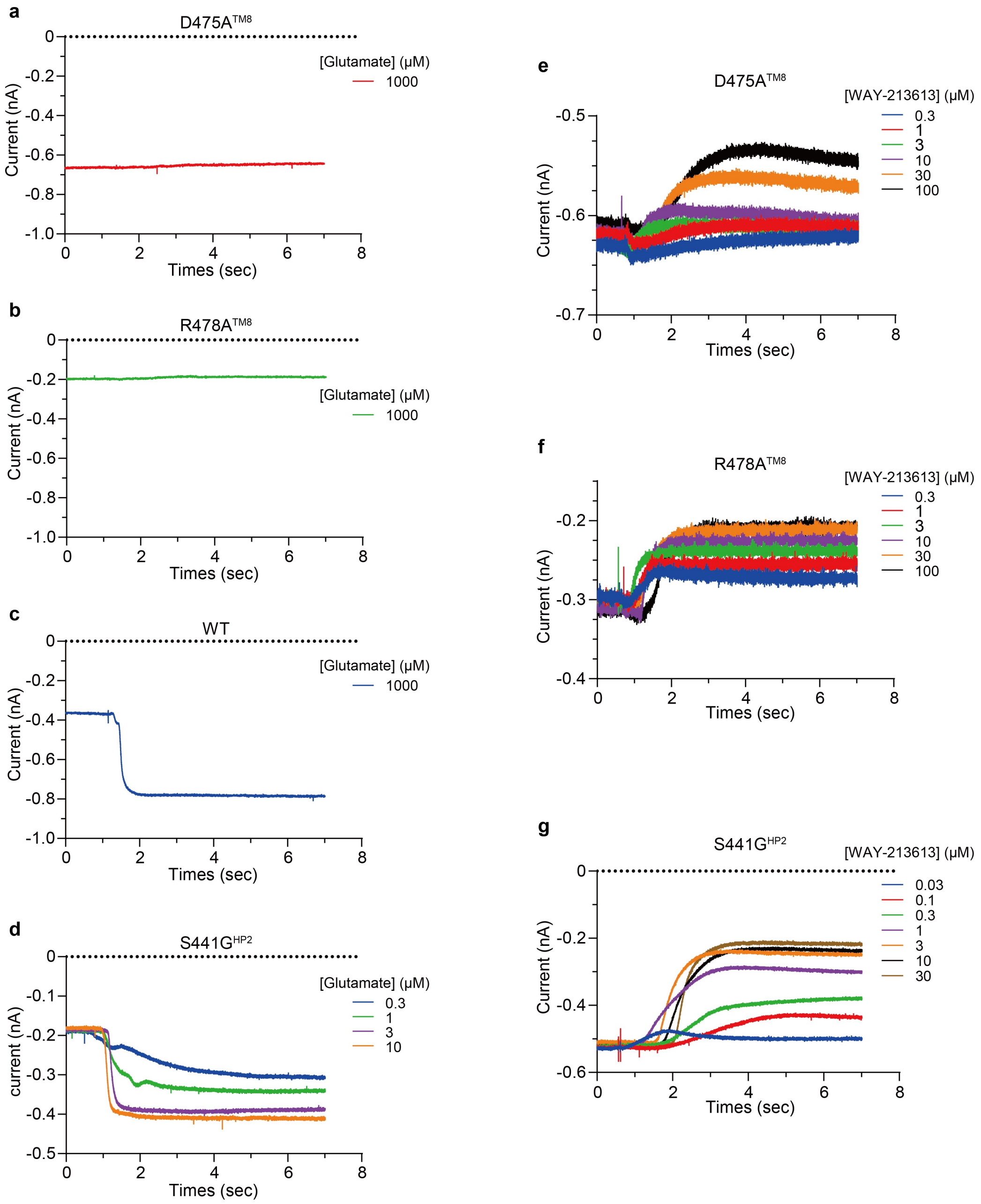

Supplementary Fig. 7 Current traces for hEAAT2 mutants D475A^{TM8}, R478A^{TM8} and S441G^{HP2} in application of glutamate or WAY-213613. a-c Currents recorded when 1000 μM glutamate are applied to the hEAAT2 mutants (D475A^{TM8} and R478A^{TM8}) or wild-type hEAAT2-expressed HEK293T cells. **d** Current traces obtained when glutamate was applied at the indicated concentrations to the hEAAT2 mutant S441G^{HP2}-expressed HEK293T cells. **e-g** Current traces obtained when WAY-213613 was applied at the indicated concentrations to

hEAAT2 mutants (D475A^{TM8}, R478A^{TM8} and S441G^{HP2})-expressed HEK293T cells. All experiments were executed at 0 mV and the experimental procedure was the same as the one described in the method.

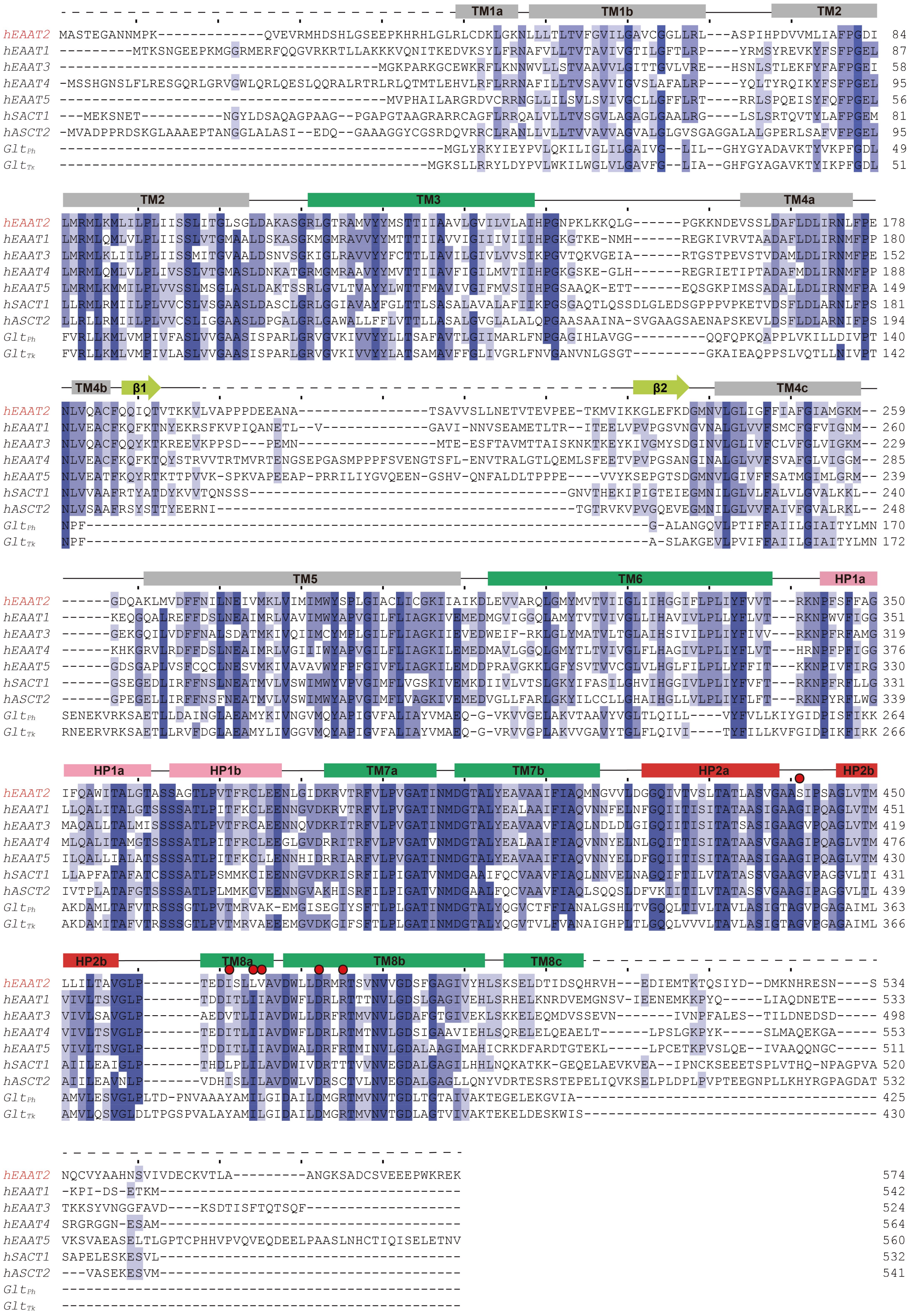

TM1a
TM1b
TM2
hEAAT2 MASTEGANNMPK-------------QVEVRMHDSHLGSEEPKHRHLGLRLCDKLGKNLLLTLTVFGVILGAVCGGLLRL---ASPIHPDVVMLIAFPGDI 84
hEAAT1 ---------MTKSNGEEPKMGGRMERFQQGVRKRTLLAKKKVQNITKEDVKSYLFRNAFVLLTVTAVIVGTILGFTLRP----YRMSYREVKYFSFPGEL 87
hEAAT3 --MGKPARKGCEWKRFLKNNWVLLSTVAAVVLGITTGVLVRE---HSNLSTLEKFYFAFPGEI 58
hEAAT4 -MSSHGNSLFLRESGQRLGRVGWLQRLQESLQQRALRTRLRLQTMTLEHVLRFLRRNAFILLTVSAVVIGVSLAFALRP----YQLTYRQIKYFSFPGEL 95
hEAAT5 ---MVPHAILARGRDVCRRNGLLILSVLSVIVGCLLGFFLRT---RRLSPQEISYFQFPGEL 56
hSACT1 ---MEKSNET----------NGYLDSAQAGPAAG--PGAPGTAAGRARRCAGFLRRQALVLLTVSGVLAGAGLGAALRG----LSLSRTQVTYLAFPGEM 81
hASCT2 -MVADPPRDSKGLAAAEPTANGGLALASI--EDQ--GAAAGGYCGSRDQVRRCLRANLLVLLTVVAVVAGVALGLGVSGAGGALALGPERLSAFVFPGEL 95
GltPh --MGLYRKYIEYPVLQKILIGLILGAIVG--LIL--GHYGYADAVKTYVKPFGDL 49
GltTk --MGKSLLRRYLDYPVLWKILWGLVLGAVFG--LIA--GHFGYAGAVKTYIKPFGDL 51
TM2
TM3
TM4a
hEAAT2 LMRMLKMLILPLIISSLITGLSGLDAKASGRLGTRAMVYYMSTTIIAAVLGVILVLAIHPGNPKLKKQLG------PGKKNDEVSSLDAFLDLIRNLFPE 178
hEAAT1 LMRMLQMLVLPLIISSLVTGMAALDSKASGKMGMRAVVYYMTTTIIAVVIGIIIVIIIHPGKGTKE-NMH------REGKIVRVTAADAFLDLIRNMFPP 180
hEAAT3 LMRMLKLIILPLIISSMITGVAALDSNVSGKIGLRAVVYYFCTTLIAVILGIVLVVSIKPGVTQKVGEIA------RTGSTPEVSTVDAMLDLIRNMFPE 152
hEAAT4 LMRMLQMLVLPLIVSSLVTGMASLDNKATGRMGMRAAVYYMVTTIIAVFIGILMVTIIHPGKGSKE-GLH------REGRIETIPTADAFMDLIRNMFPP 188
hEAAT5 LMRMLKMMILPLVVSSLMSGLASLDAKTSSRLGVLTVAYYLWTTFMAVIVGIFMVSIIHPGSAAQK-ETT------EQSGKPIMSSADALLDLIRNMFPA 149
hSACT1 LLRMLRMIILPLVVCSLVSGAASLDASCLGRLGGIAVAYFGLTTLSASALAVALAFIIKPGSGAQTLQSSDLGLEDSGPPPVPKETVDSFLDLARNLFPS 181
hASCT2 LLRLLRMIILPLVVCSLIGGAASLDPGALGRLGAWALLFFLVTTLLASALGVGLALALQPGAASAAINA-SVGAAGSAENAPSKEVLDSFLDLARNIFPS 194
GltPh FVRLLKMLVMPIVFASLVVGAASISPARLGRVGVKIVVYYLLTSAFAVTLGIIMARLFNPGAGIHLAVGG---------QQFQPKQAPPLVKILLDIVPT 140
GltTk FVRLLKMLVMPIVLASLVVGAASISPARLGRVGVKIVVYYLATSAMAVFFGLIVGRLFNVGANVNLGSGT---------GKAIEAQPPSLVQTLLNIVPT 142
TM4b
β1
β2
TM4c
hEAAT2 NLVQACFQQIQTVTKKVLVAPPPDEEANA----------------TSAVVSLLNETVTEVPEE-TKMVIKKGLEFKDGMNVLGLIGFFIAFGIAMGKM-- 259
hEAAT1 NLVEACFKQFKTNYEKRSFKVPIQANETL--V-------------GAVI-NNVSEAMETLTR--ITEELVPVPGSVNGVNALGLVVFSMCFGFVIGNM-- 260
hEAAT3 NLVQACFQQYKTKREEVKPPSD---PEMN---------------------MTE-ESFTAVMTTAISKNKTKEYKIVGMYSDGINVLGLIVFCLVFGLVIGKM-- 229
hEAAT4 NLVEACFKQFKTQYSTRVVTRTMVRTENGSEPGASMPPPFSVENGTSFL-ENVTRALGTLQEMLSFEETVPVPGSANGINALGLVVFSVAFGLVIGGM-- 285
hEAAT5 NLVEATFKQYRTKTTPVVK-SPKVAPEEAP-PRRILIYGVQEEN-GSHV-QNFALDLTPPPE----VVYKSEPGTSDGMNVLGIVFFSATMGIMLGRM-- 239
hSACT1 NLVVAAFRTYATDYKVVTQNSSS---------------------------------------GNVTHEKIPIGTEIEGMNILGLVLFALVLGVALKKL-- 240
hASCT2 NLVSAAFRSYSTTYEERNI--TGTRVKVPVGQEVEGMNILGLVVFAIVFGVALRKL-- 248
GltPh NPF---G-ALANGQVLPTIFFAIILGIAITYLMN 170
GltTk NPF---A-SLAKGEVLPVIFFAIILGIAITYLMN 172
TM5
TM6
HP1a
hEAAT2 ------GDQAKLMVDFFNILNEIVMKLVIMIMWYSPLGIACLICGKIIAIKDLEVVARQLGMYMVTVIIGLIIHGGIFLPLIYFVVT---RKNPFSFFAG 350
hEAAT1 ------KEQGQALREFFDSLNEAIMRLVAVIMWYAPVGILFLIAGKIVEMEDMGVIGGQLAMYTVTVIVGLLIHAVIVLPLLYFLVT---RKNPWVFIGG 351
hEAAT3 ------GEKGQIVVDFFNALSDATMKIVQIIMCYMPLGILFLIAGKIIEVEDWEIF-RKLGLYMATVLTGLAIHSIVILPLIYFIVV---RKNPFRFAMG 319
hEAAT4 ------KHKGRVLRDFFDSLNEAIMRLVGIIIWYAPVGILFLIAGKILEMEDMAVLGGQLGMYTLTVIVGLFLHAGIVLPLIYFLVT---HRNPFPFIGG 376
hEAAT5 ------GDSGAPLVSFCQCLNESVMKIVAVAVWYFPFGIVFLIAGKILEMDDPRAVGKKLGFYSVTVVCGLVLHGLFILPLLYFFIT---KKNPIVFIRG 330
hSACT1 ------GSEGEDLIRFFNSLNEATMVLVSWIMWYVPVGIMFLVGSKIVEMKDIIVLVTSLGKYIFASILGHVIHGGIVLPLIYFVFT---RKNPFRFLLG 331
hASCT2 ------GPEGELLIRFFNSFNEATMVLVSWIMWYAPVGIMFLVAGKIVEMEDVGLLFARLGKYILCCLLGHAIHGLLVLPLIYFLFT---RKNPYRFLWG 339
GltPh SENEKVRKSAETLLDAINGLAEAMYKIVNGVMQYAPIGVFALIAYVMAEQ-G-VKVVGELAKVTAAVYVGLTLQILL----VYFVLLKIYGIDPISFIKK 264
GltTk RNEERVRKSAETLLRVFDGLAEAMYLIVGGVMQYAPIGVFALIAYVMAEQ-G-VRVVGPLAKVVGAVYTGLFLQIVI----TYFILLKVFGIDPIKFIRK 266
HP1a
HP1b
TM7a
TM7b
HP2a
HP2b
hEAAT2 IFQAWITALGTASSAGTLPVTFRCLEENLGIDKRVTRFVLPVGATINMDGTALYEAVAAIFIAQMNGVVLDGGQIVTVSLTATLASVGAASIPSAGLVTM 450
hEAAT1 LLQALITALGTSSSSATLPITFKCLEENNGVDKRVTRFVLPVGATINMDGTALYEALAAIFIAQVNNFELNFGQIITISITATAASIGAAGIPQAGLVTM 451
hEAAT3 MAQALLTALMISSSSSTLPVTFRCAEENNQVDKRITRFVLPVGATINMDGTALYEAVAAVFIAQLNDLDLGIGQIITISITATSASIGAAGVPQAGLVTM 419
hEAAT4 MLQALITAMGTSSSSATLPITFRCLEEGLGVDRRITRFVLPVGATVNMDGTALYEALAAIFIAQVNNYELNLGQITTISITATAASVGAAGIPQAGLVTM 476
hEAAT5 ILQALLIALATSSSSATLPITFKCLLENNHIDRRIARFVLPVGATINMDGTALYEAVAAIFIAQVNNYELDFGQIITISITATAASIGAAGIPQAGLVTM 430
hSACT1 LLAPFATAFATCSSSATLPSMMKCIEENNGVDKRISRFILPIGATVNMDGAAIFQCVAAVFIAQLNNVELNAGQIFTILVTATASSVGAAGVPAGGVLTI 431
hASCT2 IVTPLATAFGTSSSSATLPLMMKCVEENNGVAKHISRFILPIGATVNMDGAALFQCVAAVFIAQLSQQSLDFVKIITILVTATASSVGAAGIPAGGVLTL 439
GltPh AKDAMLTAFVTRSSSGTLPVTMRVAK-EMGISEGIYSFTLPLGATINMDGTALYQGVCTFFIANALGSHLTVGQQLTIVLTAVLASIGTAGVPGAGAIML 363
GltTk AKDAMITAFVTRSSSGTLPVTMRVAEEEMGVDKGIFSFTLPLGATINMDGTALYQGVTVLFVANAIGHPLTLGQQLVVVLTAVLASIGTAGVPGAGAIML 366
HP2b
TM8a
TM8b
TM8c
hEAAT2 LLIITAVGLP-------TEDISLLVAVDWLLDRMRTSVNVVGDSFGAGIVYHLSKSELDTIDSQHRVH---EDIEMTKTQSIYD--DMKNHRESN----S 534
hEAAT1 VIVLTSVGLP-------TDDITLIIAVDWFLDRLRTTTNVLGDSLGAGIVEHLSRHELKNRDVEMGNSV-IEENEMKKPYQ-----LIAQDNETE----- 533
hEAAT3 VIVLSAVGLP-------AEDVTLIIAVDWLLDRFRTMVNVLGDAFGTGIVEKLSKKELEQMDVSSEVN-------IVNPFALES--TILDNEDSD----- 498
hEAAT4 VIVLTSVGLP-------TEDITLIIAVDWFLDRLRTMTNVLGDSIGAAVIEHLSQRELELQEAELT------LPSLGKPYK-----SLMAQEKGA----- 553
hEAAT5 VIVLTSVGLP-------TDDITLIIAVDWALDRFRTMINVLGDALAAGIMAHICRKDFARDTGTEKL-----LPCETKPVSLQE--IVAAQQNGC----- 511
hSACT1 AIILEAIGLP-------THDLPLILAVDWIVDRTTTVVNVEGDALGAGILHHLNQKATKK-GEQELAEVKVEA--IPNCKSEEETSPLVTHQ-NPAGPVA 520
hASCT2 AIILEAVNLP-------VDHISLILAVDWLVDRSCTVLNVEGDALGAGLLQNYVDRTESRSTEPELIQVKSELPLDPLPVPTEEGNPLLKHYRGPAGDAT 532
GltPh AMVLESVGLPLTD-PNVAAAYAMILGIDAILDMGRTMVNVTGDLTGTAIVAKTEGELEKGVIA-------------------------------------- 425
GltTk AMVLQSVGLDLTPGSPVALAYAMILGIDAILDMGRTMVNVTGDLAGTVIVAKTEKELDESKWIS------------------------------------- 430
hEAAT2 NQCVYAAHNSVIVDECKVTLA---------ANGKSADCSVEEEPWKREK 574
hEAAT1 -KPI-DS-ETKM------------------------------------- 542
hEAAT3 TKKSYVNGGFAVD----KSDTISFTQTSQF------------------- 524
hEAAT4 SRGRGGN-ESAM------------------------------------- 564
hEAAT5 VKSVAEASELTLGPTCPHHVPVQVEQDEELPAASLNHCTIQISELETNV 560
hSACT1 SAPELESKESVL------------------------------------- 532
hASCT2 ---VASEKESVM------------------------------------- 541
GltPh ---
GltTk ---

Supplementary Fig. 8 Sequence alignment of human SLC1 family transporters with its homologs. The amino acid sequences of hEAAT1 to hEAAT5, hASCT1 and hASCT2, Glt_{Ph} and Glt_{Tk} are aligned using Clustal Omega and shown on Jalview with a manual adjustment. The scaffold domain (gray), the transport domain (green), HP1 (pink) and HP2 (red), and two β strands (light green) are shown above the aligned sequences using colored rectangles or arrows. Dashed lines represent the regions which are not seen in the structure. Residues in hEAAT2 that mutated are labeled with red filled circle.

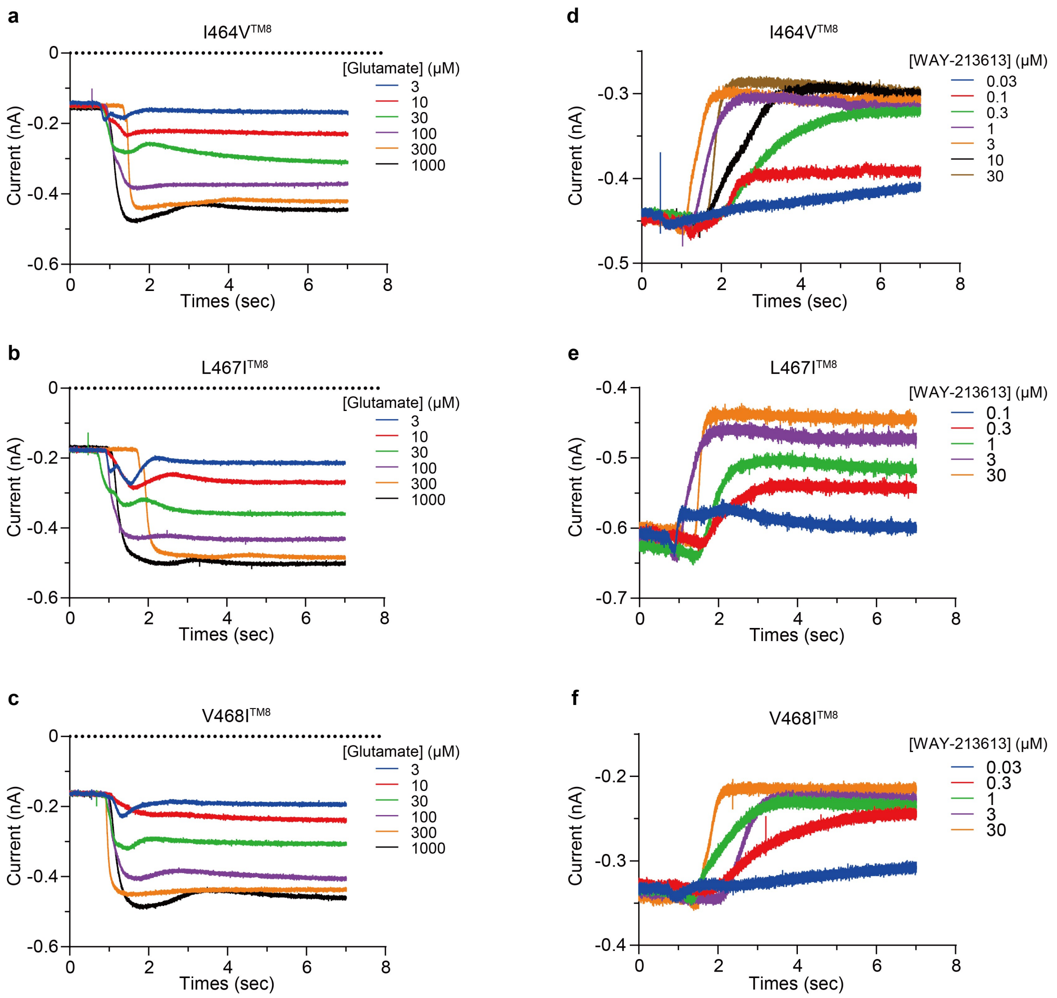

Supplementary Fig. 9 Current traces for hEAAT2 mutants I464V^{TM8}, L467I^{TM8} and V468I^{TM8} in application of glutamate or WAY-213613. a-c Current traces obtained when glutamate was applied at the indicated concentrations to the hEAAT2 mutants (I464V^{TM8}, L467I^{TM8} and V468I^{TM8})-expressed HEK293T cells. **d-f** Current traces obtained when WAY-213613 was applied at the indicated concentrations to hEAAT2 mutants (I464V^{TM8}, L467I^{TM8} and V468I^{TM8})-expressed HEK293T cells. All experiments were executed at 0 mV and the experimental procedure was the same as the one described in the method.

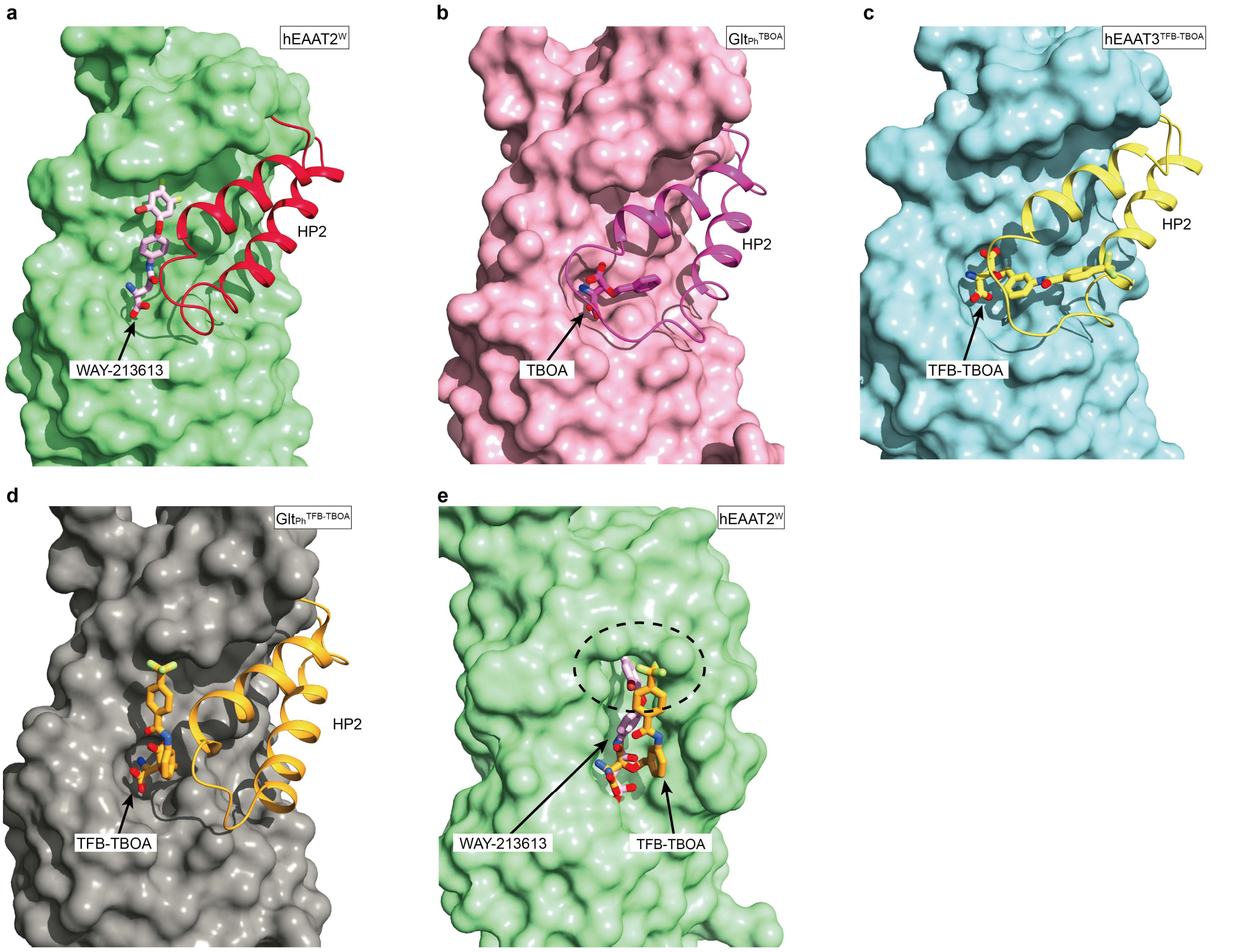

Supplementary Fig. 10 Distinct inhibitor binding pocket of EAATs and prokaryotic homologs. a Surface representation of the hEAAT2^{W} in pale green. HP2 and WAY-213613 are shown as red cartoon and pink sticks, respectively. **b** Surface representation of Glt_{Ph}^{TBOA} (PDB ID: 6X16) in pink. HP2 and TBOA are shown as magenta cartoon and sticks, respectively. **c** Surface representation of hEAAT3$^{TFB\text{-}TBOA}$ (PDB ID: 6S3Q) in cyan. HP2 and TFB-TBOA are shown as yellow cartoon and sticks, respectively. **d** Surface representation of $Glt_{Ph}^{TFB\text{-}TBOA}$ (PDB ID: 6X14) in gray. HP2 and TFB-TBOA are shown as orange cartoon and sticks, respectively. **e** Superimposed structures of hEAAT2^{W} with $Glt_{Ph}^{TFB\text{-}TBOA}$ (PDB ID: 6X14). WAY-213613 and TFB-TBOA are shown as pink and orange sticks, respectively.

Supplementary Table

Supplementary Table 1 Cryo-EM data collection, refinement and validation statistics.

	hEAAT2^{Glu} (EMDB-33407) (PDB 7XR4)	hEAAT2^{W} (EMDB-33408) (PDB 7XR6)
**Data collection and processing**		
Magnification	105,000 ×	105,000 ×
Voltage (kV)	300	300
Electron exposure (e^{-}/Å^2)	60	60
Defocus range (μm)	−1.2 ~ −2.2	−1.2 ~ −2.2
Pixel size (Å)	1.04	1.04
Symmetry imposed	C3	C3
Initial particle images (no.)	1,509,287	119,277
Final particle images (no.)	78,254	83,977
Map resolution (Å)	3.4	3.4
FSC threshold	0.143	0.143
Map resolution range (Å)	3.0 ~ 5.0	3.0 ~ 5.0
**Refinement**		
Initial model used (PDB code)	6S3Q	hEAAT2^{Glu}
Model resolution (Å)	3.4	3.5
FSC threshold	0.5	0.5
Map sharpening *B* factor (Å^2)	156	118
Model composition		
Non-hydrogen atoms	10,026	10,199
Protein residues	1,278	1,272
Ligands	27	30
B factors (Å^2)		
Protein	33.55	45.54
Ligand	34.04	51.36
R.m.s. deviations		
Bond lengths (Å)	0.006	0.004
Bond angles (°)	0.665	0.600
Validation		
MolProbity score	1.50	1.58
Clashscore	9	9
Poor rotamers (%)	0.29	0.00
Ramachandran plot		
Favored (%)	97.85	97.53
Allowed (%)	2.15	2.47
Disallowed (%)	0.00	0.00

Source data of Supplementary Fig.1c

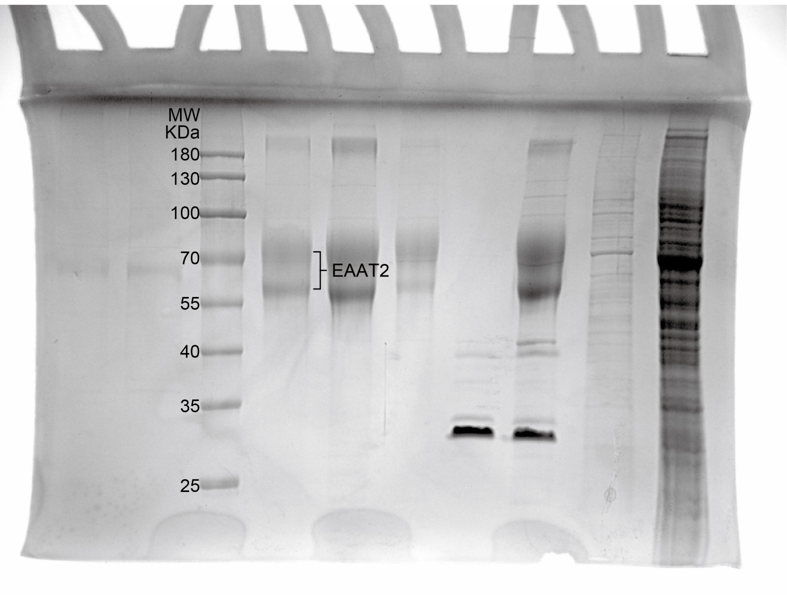

The uncropped scan of gel in Supplementary Fig.1c.
